# Microwave Assisted Dyeing of Polyester Fabrics with Disperse Dyes

**DOI:** 10.3390/molecules180911033

**Published:** 2013-09-09

**Authors:** Saleh Mohammed Al-Mousawi, Morsy Ahmed El-Apasery, Mohamed Hilmy Elnagdi

**Affiliations:** 1 Chemistry Department, Faculty of Science, Kuwait University, P.O. Box 5969, Safat 13060, Kuwait; E-Mail: shelmy1941@yahoo.com; 2 Dyeing, Printing and Textile Auxiliaries Department, Textile Research Division, National Research Centre, Dokki, Giza 12622, Egypt

**Keywords:** polyester fabrics, disperse dyes, thienobenzochromene, microwave heating

## Abstract

Dyeing of polyester fabrics with thienobenzochromene disperse dyes under conventional and microwave heating conditions was studied in order to determine whether microwave heating could be used to enhance the dyeability of polyester fabrics. Fastness properties of the dyed samples were measured. All samples dyed with or without microwave heating displayed excellent washing and perspiration fastness. The biological activities of the synthesized dyes against Gram positive bacteria, Gram negative bacteria, yeast and fungus were also evaluated.

## 1. Introduction

The energy of microwave photons is quite low compared with the chemical bond energies and, thus, microwaves do not directly affect the molecular structure of a compound and they do not change the electronic configuration of atoms [1,2]. When materials are exposed to microwave irradiation, they are affected differently, some materials reflect the microwaves, others transmit them and some absorb them. Microwave heating is an alternative technique to conventional heating for fast, effective and uniform heating. Because microwave energy easily penetrates all of the particles of materials, the heating takes place instantly and uniformly [3], which, in turn, eliminates the problems that could occur during conventional heating [4,5,6,7,8,9,10,11,12]. In the disperse dyeing of polyester fabrics the use of microwave heating promotes dye exhaustion and increases the dyeing rates [13]. Despite the large number of reports on the utility of aminothienobenzochromene dyes in the dye industry [14,15,16,17,18], to our knowledge, their corresponding arylazothienobenzochromenes have never been reported as potential monoazo disperse dyes. In view of these findings, and in continuation of our previous study on the synthesis of a variety of aminothienobenzochromene dyes [19], we now report the synthesis of some arylazothienobenzochromenes dyes and their application as disperse dyes for dyeing polyester fabrics using microwave heating to enhance the dyeability of polyester fabrics and to investigate both of the colour and fastness properties of the dyed materials. The biological activities of the synthesized dyes against Gram negative bacteria, Gram positive bacteria, yeast and filamentous fungi were evaluated.

## 2. Results and Discussion

### 2.1. Chemistry

**Scheme 1:**
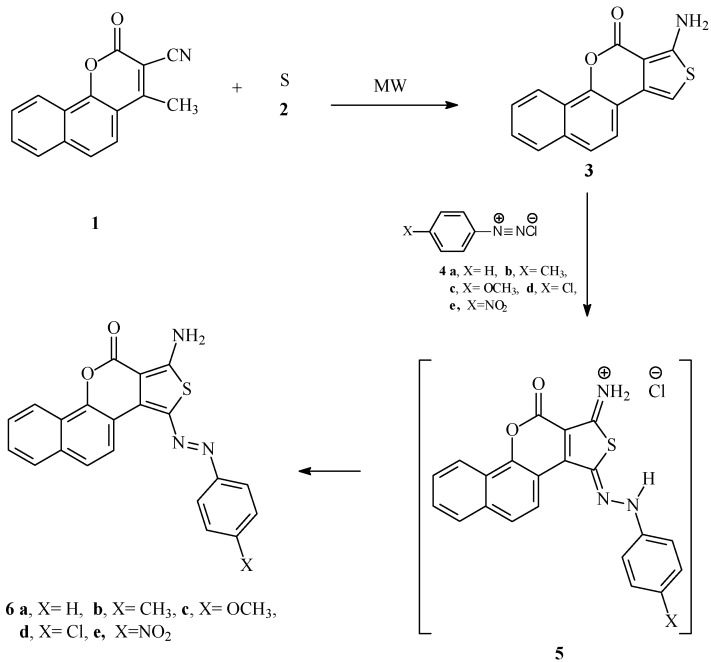
Preparation of monoazo disperse dyes **6a**–**e**.

In conjunction with our interest in developing efficient routes to polyfunctional heteroaromatics as potential disperse dyes for polyester fabrics; we report here results of the reactivity of thienobenzochromene toward some aryldiazonium chlorides. As we have placed emphasis in the last few years on adopting microwave heating as a suitable alternative to conventional heating [19], we have utilized heating in a direct beam microwave oven, for our syntheses. We observed that reaction of 4-methyl-2-oxo-2*H*-benzo[h]chromene-3-carbonitrile **1** with elemental sulfur in presence of morpholine as basic catalyst in a microwave oven as an energy source at 130 °C for 5 min, thienobenzochromene **3** is produced in 93% yield (Scheme 1).

The thienobenzochromene **3** was then coupled with aryldiazonium chlorides to afford aminoarylazo thienobenzochromene disperse dyes **6a**–**e**. These compounds are most likely formed *via* intermediacy of compound **5**.

### 2.2. Dyeing

Disperse dyes **6a**–**e** were applied to polyester fabrics at 2% (dye shade), using the high temperature (HT) dyeing method at 130 °C. Orange to violet color shades were obtained (Table 1). The dyeing properties on the polyester fabrics were evaluated in terms of their fastness properties (e.g., fastnesses to washing and perspiration). The dyeing color on polyester fabrics is expressed in terms of CIELAB values (Table 1 and Table 2), and the following CIELAB coordinates were measured: lightness (L*); chroma (C*); hue angle (*h*) from 0 to 360°; a*, whose value represents the degree of redness (positive) and greenness (negative); and b*, whose value represents the degree of yellowness (positive) and blueness (negative). Hue *h* is one of the three basic characters for color. The *h* values in Table 1 show that almost all of the dyed polyester samples expressed the same hue, except dye **6e** as a violet color. In general, the color hues of the disperse dye **6e** on the polyester fabric shifted to the bluish directions; this was indicated by the negative value of b* = −14.73 and −16.78 (yellow–blue axis). The positive values of a* and b* indicated that the color hues of the disperse dyes **6a**–**d** on the polyester fabric shifted in the reddish direction.

**Table 1:** Shade and optical measurements of the azo disperse dyes on the polyester fabrics using conventional dyeing method.

Dye No	Color on polyester (2% shade)	L*	a*	b*	C*	h*	*K/S*
6a	Brown	40.81	22.38	25.06	33.60	48.24	19.99
6b	Orange	62.30	47.93	45.13	65.83	43.28	8.11
6c	Dark orange	48.52	49.53	45.28	67.10	42.43	23.6
6d	Orange	60.77	49.43	34.74	60.42	35.10	6.66
6e	Violet	43.82	36.23	−14.73	39.11	337.87	6.89

Microwave heating is quite different from conventional heating, where the heat must diffuse in the media from the surface of the material. In volumetric heating, the materials can absorb microwave energy directly and internally and convert it into heat. This leads to advantages such as rapid, controlled, selective and uniform heating [2]. Moreover, it is known that microwave heating enhances diffusion of organic molecules in polymers and this can increase the fixing rate of dyes into the polymeric textiles [20]. The colorimetric parameters and the colour strength (K/S) values obtained for the microwave dyed fabrics and the conventional dyed fabrics are given in Table 2. In the context of the colorimetric data recorded for the samples, Table 2 shows that the microwave dyed fabric with disperse dyes **6a**–**e** are darker (ΔL* = −18.74, −4.87, −19.7, −8.61 and 1.17), slightly less red (Δa* = −9.66, 1.13, −22.83, 6.7 and −1.38), less yellow (Δb* = −8.87, 6.84, −21.56, 8.53 and −2.05) and slightly less saturated (ΔC* = −13.01, 5.75, −31.39, 10.46 and −0.43) than the conventional samples. The K/S values are ranged from 7.03 to 39.47 and 6.89 to 23.60 for the microwave dyed fabric and the conventional dyed fabric, respectively, All of these results indicate that the microwave dyed samples are better than the conventional samples in a very short dyeing time, These results in this study support the findings of the other researchers, like Oner *et al.* [3], and Haggag *et al.* [4], who showed that the microwave dyeing method is better than the conventional method due to shorter dyeing time, energy saving and better dye uptake.

**Table 2:** Shade and optical measurements of the azo disperse dyes on the polyester fabrics using microwave dyeing method.

Dye No	Color on polyester (2% shade)	L*	a*	b*	C*	h*	*K/S*
6a	Dark brown	22.07	12.72	16.19	20.59	51.84	33.24
6b	Dark orange	57.43	49.23	51.97	71.58	46.55	15.31
6c	Brownish orange	28.82	26.7	23.72	35.71	41.61	39.47
6d	Dark orange	52.16	56.13	43.27	70.88	37.63	18.77
6e	Violet	44.99	34.85	−16.78	38.68	334.28	7.03

The fastness properties for the conventionally dyed and the microwave dyed fabrics given in Table 3 and Table 4 show that both dyed fabrics have excellent fastness levels to washing and perspiration.

**Table 3:** Fastness properties of monoazo disperse dyes on polyester fabrics using conventional dyeing method.

Dye No	Wash fastness			Perspiration fastness					
				Alkaline			Acidic		
	Alt	SC	SW	Alt	SC	SW	Alt	SC	SW
**6a**	5	5	5	5	5	5	5	5	5
**6b**	5	5	5	5	5	5	5	5	5
**6c**	5	5	5	5	5	5	5	5	5
**6d**	5	5	5	5	5	5	5	5	5
**6e**	5	5	5	5	5	5	5	5	5

Alt = alteration; SC = staining on cotton; SW = staining on wool.

**Table 4:** Fastness properties of monoazo disperse dyes on polyester fabrics using microwave dyeing method.

Dye No	Wash fastness			Perspiration fastness					
				Alkaline			Acidic		
	Alt	SC	SW	Alt	SC	SW	Alt	SC	SW
**6a**	5	5	5	5	5	5	5	5	5
**6b**	5	5	5	5	5	5	5	5	5
**6c**	5	5	5	5	5	5	5	5	5
**6d**	5	5	5	5	5	5	5	5	5
**6e**	5	5	5	5	5	5	5	5	5

Alt = alteration; SC = staining on cotton; SW = staining on wool.

### 2.3. Absorption Spectral Characteristics

The absorption maxima of the dyes **6a**–**e** recorded in various solvents ranged from 345 to 660 nm and are shown in Table 5.

**Table 5:** Influence of solvent on ***λ***_max_ (nm) of dyes.

Dye No	X	Absorption *λ*_max_ (nm)		
		Methanol	Chloroform	DMF
**6a**	*p*-H	345	353	349
**6b**	*p*-CH_3_	481	482	493
**6c**	*p*-OCH_3_	486	489	499
**6d**	*p*-Cl	487	487	513
**6e**	*p*-NO_2_	525	523	660

Colour shifts observed in these dyes are in accordance to variations resulting from changes in substituents in the diazonium component. Thus, the incorporation of an electron-donating substituent at the *para-*position of the arylzono moiety results in a significant bathochromic shift, the greater the strength of the electron-donating substituent, the more significant the bathochromic shift. Thus, the dyes **6b** and **6c** (*λ*_max_ 481 and 486 nm), were characterized by significant bathochromic shifts compared with dye **6a** (*λ*_max_ 345 nm) (Δ*λ*_max_ = 136 and 141 nm), attributable to the CH**_3_** and OCH**_3_** groups, respectively. The observed bathochromism upon introduction of electron donor methyl and methoxy groups is in agreement with the theory. The densities of electrons that are added to delocalization are increased due to electron donor properties of the methyl and methoxy groups. Bathochromic shifts occur due to this effect. The spectroscopic data also demonstrate that the incorporation of NO**_2_** group in dye **6e** gave a better exhaustion and noticeable depth of dye color (Δ*λ*_max_ = 180 nm).

### 2.4. Antimicrobial Activities

The antimicrobial activities of the synthesized disperse dyes were screened against selected bacteria and fungi by the agar well diffusion method and their inhibition zones diameters, given in Table 6, reveal that the tested dyes **6a**–**e** showed positive antimicrobial activities. Dye **6a**–**e** showed no activities against the fungus that used in the study. The tested dye **6a** displayed strong inhibitory effects on the growth of *S. cerevisiae* which showed inhibition zones exceeding 6 mm compared to the reference chemotherapeutic flumox. Moreover dyes **6a**, **6c** and **6e** revealed higher activities against the Gram negative than those recorded for the Gram positive bacteria as revealed by the diameters of their inhibition zones.

**Table 6:** Diameter of the zones of inhibition of the tested disperse dyes against Gram positive, Gram negative bacteria, yeast and fungi.

Dye No.	Inhibition zone diameter			
	*G+ve* bacteria	*G-ve* bacteria	Yeast	Fungus
	*B. subtilis*	*E. coli*	*S. cerevisiae*	*A. niger*
**6a**	-	4	6	-
**6b**	-	-	2	-
**6c**	1	2	2.5	-
**6d**	-	-	0.5	-
**6e**	2	2.5	4.5	-
Flumox *	4	5	4	

(-) no inhibition; * Flumox: reference antibacterial (100 mg·mL^−1^).

## 3. Experimental

### 3.1. General

Melting points were recorded on a Gallenkamp apparatus. IR spectra were recorded using KBr pellets on a JASCO FTIR-6300 FT-IR spectrophotometer. ^1^H- and ^13^C-NMR spectra were recorded on Bruker DPX 400 MHz or Avance II 600 MHz super-conducting NMR spectrometers with proton spectra measured at 400, 600 MHz and carbon spectra at 100 and 150 MHz, respectively. Mass spectra were measured on a high resolution GC/MS DFS-Thermo. Microanalyses were performed on Elementar-Vario Micro cube Analyzer. The dyeing of polyester fabrics were conducted using LINITEST+Laboratory High Temperature Dyeing and Fastness System (ATLAS MTT GmbH, Altenhasslau, Germany). The colorimetric parameters of the dyed polyester fabrics were determined on a reflectance spectrophotometer (UltraScan PRO D65, HunterLab, Reston, VA, USA).

### 3.2. 17-Amino-11-oxa-16-thia-cyclopenta[a]phenanthren-12-one (**3**)

A mixture of compound **1** (2.35 g, 10 mmol), sulfur (0.32 g, 10 mmol) and dioxane (2 mL) in the presence of few drops of morpholine, was irradiated by focused microwave at 130 °C for 5 min. Completion of the reactions was monitored by TLC. The build-up of pressure in the closed reaction vessel was carefully monitored. After the irradiation, the reaction tube was cooled with high-pressure air through an inbuilt system in the instrument until the temperature had fallen below 50 °C. The mixtures were cooled and then poured into ice-water. The solids that formed were collected by using filtration and crystallized from dioxane to give compound **3** as a green powder (93%), mp 262–264 °C, IR (KBr): 3410, 3296 (NH_2_), 1679 (CO) cm^−1^. ^1^H-NMR (DMSO-*d_6_*): δ = 6.97 (1H, s, 1-H); 7.57–7.65 (m, 2H, arom-H), 7.75 (d, 1H, *J* = 8.4 Hz, arom-H), 7.84 (s. 2H, NH_2_, D_2_O-exchangeable), 7.93 (t, 2H, *J* = 8.0 Hz, arom-H), 8.24 (d, 1H, *J* = 8.0 Hz, arom-H). ^13^C-NMR (DMSO-*d_6_*): δ = 166.6 (CO), 158.5, 145.6, 139.2, 131.3, 127.9, 127.1, 127.0, 123.9, 123.2, 121.3, 121.0, 113.1, 97.9, 97.6. MS (EI) *m/z =* 267 (M]^+^, 100), HRMS: *m/z* (EI) for C15H9NO2S; calcd. 267.0384; found: 267.0384.

### 3.3. Reaction of Compounds **3** with Aromatic Diazonium Salts

A solution of each aryldiazonium chloride (10 mmol. prepared as described earlier [18]) was added at 0 °C to a solution of **3** (10 mmol) in acetic acid (50 mL) containing sodium acetate (0.60 g). The reaction mixture was stirred at room temperature for 1 hr and the solid product was collected by filtration and crystallized from DMF/ethanol (3:1).

*3-Amino-1-phenylazo)thieno[3,4-c][1]benzopyran-4-one* (**6a**). Brown crystals (81%), mp >300 °C (lit. [18], mp >300 °C). IR: ν_max_/cm^−1^ 3391 and 3274 (NH_2_), 1699 (CO); ^1^H-NMR (DMSO-*d*_6_): δ_H_ 7.07 (br s, 2H, NH_2_), 7.40 (t, 1H, *J* = 7.4 Hz, phenyl-H), 7.55 (t, 2H, *J* = 7.6 Hz, phenyl-H), 7.71 (t, 2H, *J* = 7.2 Hz, arom-H), 7.76 (d, 2H, *J* = 8.8 Hz, phenyl-H). 7.95 (d, 1H, *J* = 8.9 Hz, arom-H), 8.04 (t, 1H, *J* = 7.2 Hz, arom-H). 8.37 (t, 1H, *J* = 7.2 Hz, arom-H), 8.93 (d, 1H, *J* = 8.8 Hz, arom-H). ^13^C-NMR (DMSO-*d_6_*): δ_C_ 169.55 (CO), 157.4, 153.2, 149.8, 147.4, 137.6, 135.1, 131.6, 130.5, 129.9, 129.0, 128.4, 125.3, 123.8, 123.0, 122.7, 113.7, 104.8, 102.3. MS: *m/z* (100%) 371 [M^+^]. Anal. Calcd for C_21_H_13_N_3_O_2_S: C, 67.91; H, 3.52, N, 11.31; S, 8.63. Found C, 67.96; H, 3.23; N, 11.06; S, 8.50%.

*3-Amino-1-(p-tolylazo)-thieno[3,4-c][1]benzopyran-4-one* (**6b**). Orange powder (75%), IR (KBr): 3397 and 3275 (NH_2_), 1702 (CO) cm^−1^; ^1^H-NMR (DMSO-*d*_6_): δ = 2.39 (s, 3H, CH_3_), 7.34 (d, 2H, *J* = 7.8 Hz, arom-H), 7.65-7.71 (m, 6H, arom-H and NH_2_), 7.91 (d, 1H, *J* = 9.0 Hz, arom-H), 8.01 (t, 1H, *J* = 7.2 Hz, arom-H), 8.34 (t, 1H, *J* = 5.4 Hz, arom-H), 8.88 (d, 1H, *J* = 9.0 Hz, arom-H). MS (EI) *m/z =* 385 (M]^+^, 100), HRMS: *m/z* (EI) for C22H15N3O2S; calcd. 385.0871; found: 385.0871.

*3-Amino-1-(4-methoxyphenyl)-thieno[3,4-c][1]benzopyran-4-one* (**6c**). Deep orange powder (70%), mp 283–284 °C. IR (KBr): 3392 and 3277 (NH_2_), 1708 (CO) cm^−1^. ^1^H-NMR (DMSO-*d_6_*): δ = 3.56 (s, 3H, OCH_3_), 7.02 (d, 2H, *J* = 9.0 Hz, arom-H), 7.62–7.80 (m, 7H, arom-H and NH_2_), 7.92 (d, 1H, *J* = 7.2 Hz, arom-H), 8.25 (d, 1H, *J* = 9.0 Hz, arom-H), 8.74 (d, 1H, *J* = 9.0 Hz, arom-H). MS (EI) *m/z =* 401 (M]^+^, 17). HRMS: *m/z* (EI) for C22H15N3O3S; calcd. 401.0821; found: 401.0821.

*3-Amino-1-(4-chlorophenylazo)thieno[3,4-c][1]benzopyran-4-one* (**6d**). Orange crystals (78%), mp. >300 °C (lit. [18], mp >300 °C). IR: ν_max_/cm^−1^ 3395 and 3280 (NH_2_), 1699 (CO). ^1^H-NMR (CDCl_3_): δH 7.58 (d, 2H, *J* = 8.4 Hz, 4-chlorophenyl-H), 7.70–7.76 (m, 4H, arom-H and 4-chlorophenyl-H), 7.93 (d, 1H, *J* = 8.8 Hz, arom-H), 8.03–8.05 (m, 1H, arom-H), 8.32 (br s, 2H, NH_2_, D_2_O exchangeable), 8.36–8.38 (m, 1H, arom-H). 8.88 (d, 1H, *J* = 8.8 Hz, arom-H). MS (EI) *m/z =* 405 (M]^+^, 100), Anal. Calcd for C_21_H_12_ClN_3_O_2_S: C, 62.14; H, 2.98; N, 10.35; S, 7.90. Found C, 62.21; H, 3.17; N, 10.46; S, 7.67%. HRMS: calcd. 405.0336; found: 405.0336.

*3-Amino-1-(4-nitrophenylazo)thieno[3,4-c][1]benzopyran-4-one* (**6e**). Violet crystals (74%), mp. > 300 °C (lit. [18], mp >300 °C). IR: ν_max_/cm^−1^ 3387 and 3270 (NH_2_), 1703 (CO); ^1^H-NMR (DMSO-*d*_6_): δ_H_ 7.12 (br s, 2H, NH_2,_ D_2_O exchangeable), 7.72–7.73 (m, 2H, arom-H), 7.84 (d, 2H, *J* = 9 Hz, 4-nitrophenyl-H), 7.94 (d, 1H, *J* = 8.8 Hz, arom-H), 8.06 (m, 1H, arom-H), 8.32 (d, 2H, *J* = 9 Hz, 4-nitrophenyl-H), 8.38 (d, 1H, *J* = 7 Hz arom-H), 8.90 (d, 1H, *J* = 8.8 Hz, arom-H). MS (EI) *m/z =* 416 (M]^+^, 100). Anal. Calcd for C_21_H_12_N_4_O_4_S: C, 60.57; H, 2.90; N, 13.15; S, 7.70. Found C, 60.22; H, 3.14; N, 13.18; S, 7.27%. HRMS: calcd. 416.0573; found: 416.0573.

### 3.4. High Temperature (HT) Dyeing Method

#### 3.4.1. Materials

Polyester 100% (150 130 g/m^2^, 70/2 denier) was used. The fabric was treated before dyeing with a solution containing non-ionic detergent (Sera Wash M-RK, 5 g/L) and sodium carbonate (2 g/L) in a ratio of 50:1 at 60 °C for 30 min, then thoroughly washed with water and air dried at room temperature.

#### 3.4.2. Dyeing

##### 3.4.2.1. Conventional Dyeing

The dye baths were prepared from the dye (2% weight of fabric) to a final liquor of 50:1, w/w. The pH value of the bath was adjusted to 4.5–5 with acetic acid (10%) in the presence of a 1:1 ratio of the dispersing agent (Sera Gal P-LP). The temperature was raised to 130 °C at the rate of 7 °C/min, and dyeing continued for 60 min. After dyeing, the fabrics were thoroughly washed and then subjected to a surface reduction clearing [(2 g NaOH + 2 g sodium hydrosulphite)/L]. The samples were heated in this solution for 30 min. at 80 °C and then thoroughly washed and air-dried.

##### 3.4.2.2. Microwave Dyeing

The dye baths were prepared from the dye (2% weight of fabric) to a final liquor of 50:1, w/w. The pH value of the bath was adjusted to 4.5–5 with acetic acid (10%) in the presence of a 1:1 ratio of the dispersing agent (Sera Gal P-LP). The temperature was raised to 130 °C in focused microwave oven (CEM Explorer Microwave, Matthews, NC, USA) and dyeing continued for 20 min. After dyeing, the fabrics were thoroughly washed and then subjected to a surface reduction clearing [(2 g NaOH + 2 g sodium hydrosulphite)/L]. The samples were heated in this solution for 30 min. At 80 °C and then thoroughly washed and air-dried.

### 3.5. Color Measurements and Analyses

#### 3.5.1. Color Measurements

The colorimetric parameters of the dyed polyester fabrics (Table 1 and Table 2) were determined on a reflectance spectrophotometer. The color yields of the dyed samples were determined by using the light reflectance technique performed on an UltraScan PRO D65 UV/VIS Spectrophotometer. The color strengths, expressed as *K/S* values, were determined by applying the Kubelka-Mink equation as follows:

K/S = [(1 − R)^2^/2R] − [(1 − R_o_)^2^/2R_o_] (1)

where *R* = decimal fraction of the reflectance of the dyed fabric; *R_o_* = decimal fraction of the reflectance of the undyed fabric; *K* = absorption coefficient; *S* = scattering coefficient.

#### 3.5.2. Fastness Testing

##### 3.5.2.1. Fastness to Washing

After washing using 2 g/L of the nonionic detergent Hostapal CV at 80 °C for 15 min, the dyed fabrics were tested by using standard methods [21]. A specimen of dyed polyester fabric was stitched between two pieces of undyed cotton and wool fabrics, all of equal length, and then washed at 95 °C for 30 min. The staining on the undyed adjacent fabrics was assessed according to the following gray scale: 1—poor, 2—fair, 3—moderate, 4—good, 5—excellent.

##### 3.5.2.2. Fastness to Perspiration

The samples were prepared by stitching a piece of dyed polyester fabric between two pieces of cotton and wool fabrics, all of equal length, and then immersed in the acid or alkaline solution for 30 min. The staining on the undyed adjacent fabrics was assessed according to the following gray scale: 1—poor, 2—fair, 3—moderate, 4—good, 5—excellent. The acid solution (pH = 4.5) contains sodium chloride (10 g/L), sodium dihydrogen orthophosphate (1 g/L) and histidine monohydrochloride (0.25 g/L). The alkaline solution (pH = 9.5) contains sodium chloride (10 g/L), disodium orthophosphate (1 g/L) and histidine monohydrochloride (0.25 g/L).

### 3.6. Antimicrobial ActivitiesTest

The antimicrobial activities of some disperse dyes were tested using the agar-well diffusion technique [22] against five different microbial cultures obtained from the National Research Centre (Giza, Egypt). Pure cultures of *Bacillus subtilis* (Gram positive bacterium), *Escherichia coli* (Gram negative bacterim), *Saccharomyces cerevisiae* (yeast) and *Asparigillus niger* (fungus) were used in the test. An aliquot of 0.1 mL of each bacterial strain was inoculated and spread on nutrient agar (NA) while 0.1 mL of each yeast and filamentous fungi were spread on potato dextrose agar (PDA). The inoculated plates were supplied with 100 µL of each of the tested dyes with a total final concentration of 100 mg mL^−1^. The dyes were included in 4 mm wells produced by sterile cork borer. The NA plates were incubated at 37 °C for 24 h while PDA plates were incubated at 25 °C for 24–48 h. For reference drugs 100 µL with a total final concentration of 100 mg mL^−1^ of flumox (EIPICO, Cairo, Egypt) was used as antibacterial drug reference also scored against yeast. 

## 4. Conclusions

In summary, a series of disperse dyes were synthesized based on the arylazothienobenzochromene moiety. The dyes produced in this manner were then applied to polyester fabrics using both conventional and microwave heating dyeing methods at 130 °C. The fabrics dyed by both methods displayed excellent fastness levels to washing and perspiration. Finally, the biological activities of the synthesized disperse dyes against Gram positive bacteria, Gram negative bacteria, yeast and fungus were discussed.
